# The Management of Glucocorticoid Therapy in Liver Failure

**DOI:** 10.3389/fimmu.2019.02490

**Published:** 2019-10-23

**Authors:** Ran Xue, Qinghua Meng

**Affiliations:** ^1^Department of Critical Care Medicine of Liver Disease, Beijing You-An Hospital, Capital Medical University, Beijing, China; ^2^Key Laboratory of Carcinogenesis and Translational Research (Ministry of Education), Department of Gastrointestinal Oncology, Peking University Cancer Hospital & Institute, Beijing, China

**Keywords:** glucocorticoids, liver failure, timing, dosing, mechanism

## Abstract

Liver failure is characterized by rapid progression and high mortality. Excessive systemic inflammation is considered as the trigger of liver failure. Glucocorticoids (GCs) can rapidly suppress excessive inflammatory reactions and immune response. GCs have been applied in the treatment of liver failure since the 1970s. However, until now, the use of GCs in the treatment of liver failure has been somewhat unclear and controversial. New research regarding the molecular mechanisms of GCs may explain the controversial actions of GCs in liver failure. More results should be confirmed in a larger randomized clinical trial; this can aid the discovery of better definitions in terms of treatment schedules according to different clinical settings. Meanwhile, the timing and dosing of GCs in the treatment of liver failure should also be explored.

## Background

Liver failure is a life-threatening clinical syndrome with heterogeneous etiology that can cause serious disorders, such as coagulation disorders, icteria, hepatic encephalopathy (HE), and ascites (1, 2). Despite significant advances in artificial liver support system (ALSS) and liver transplantation (LT), these techniques are still difficult to apply more widely due to many restrictions, such as the amount of plasma, the limitation of liver donors, and the patient's economic situation, and so the mortality of liver failure is still high (3–5). It is therefore essential to develop more effective therapies for liver failure.

Glucocorticoids (GCs) have been applied to the clinical treatment of liver failure for many years. The first paper on GCs therapy for liver failure was published in the 1960s. Nowadays, many basic and clinical studies have explored the feasibility of GCs treatment in liver failure (6–12), but they remain inconclusive for the application of GCs treatment in liver failure.

## The Applied Status of GCs Therapy in Liver Failure

Among the different liver diseases, the most authoritative clinical indication of GCs therapy is autoimmune hepatitis (AIH) (7). However, in patients with suspected drug-induced AIH who are undergoing GCs therapy, withdrawal of treatment once the liver injury has resolved should be accompanied by close monitoring (13). A recent report from APASL ACLF Research Consortium Working Party defined the histopathological, clinical spectrum, and role of GCs therapy in patients with AIH-ACLF. It was shown that early stratification to LT or GCs therapy (hepatic encephalopathy in ≥F3, MELD>27) would improve outcomes and reduce ICU stay in patients with AIH-ACLF (14).

GCs therapy is also recommended as a first-line treatment strategy in patients with severe alcoholic hepatitis, hepatic encephalopathy, or maddrey discriminant function ≥32 (6). Meanwhile, GCs would not increase occurrence of or mortality from bacterial infections in patients with severe alcoholic hepatitis (15). However, a recent meta-analysis showed that it could not determine whether GCs had a positive or negative effect on people with alcoholic liver disease because available data were still insufficient to produce robust results, trials were small, and the included participants differed in severity of disease (16).

Drug-induced liver failure requires evidence of immunopathogenicity to reverse the condition through GCs blocking immune responses. A recent study showed that short-term use of GCs was strongly recommended for severe DILI patients with hyperbilirubinemia (TBil >243 μmol/L) (17). However, Wan et al. found that prednisone was not beneficial for the treatment of severe drug-induced liver injury (18). The newest EASL clinical practice guidelines for drug-induced liver injury consider how GCs are often given when all else fails to procedure results (19). Early trials of GCs therapies, for all forms of ALF, demonstrated limited benefits (10, 20). GCs are also applied to treat drug-induced cholestatic hepatitis, especially in patients with allergic manifestations such as fever, eosinophilia, and rash. Liver injury caused by antiepileptic drugs are commonly related to features of hypersensitivity and may respond to GCs (21).

There exist significant differences in the etiology of liver diseases between the East and West. HBV is the leading cause of chronic liver disease in the Asia-Pacific region, including China and India (2). HBV-activated immune response and immune pathology caused by liver cell inflammation and necrosis are the initiated factors of liver failure. Although a large number of studies reported that GC therapy is effective in liver failure (22, 23), GC therapy is only recommended for the treatment of early stages of liver failure, and there is little evidence to support its effectiveness.

However, with the arrival of nucleoside analogs (NAs), more and more guidelines have recommended NAs to be used in patients with acute exacerbation of chronic HBV infection. The early combined use of NAs and GCs could be a good option to reverse the potential deterioration in patients with HBV-related liver failure. A recent study reported that early combination therapy with corticosteroid and NAs induces rapid resolution of inflammation in ALF due to transient HBV infection (24). It has been shown that with sufficient doses of NAs, GCs cannot affect the replication of HBV (12). However, Huang et al. (12) investigated retrospectively the efficacy of GCs in patients with hepatitis B virus-related acute-on-chronic liver failure (HBV-ACLF). It was indicated that GC treatment did not improve transplant-free survival in patients with HBV-ACLF.

It is not rare for GCs to be abused in the treatment of liver failure as “reduced bilirubin drugs.” Therefore, its use in terms of liver failure therapy should not be exaggerated, although some patients with liver failure can indeed benefit from GCs therapy. As a “double-edged sword,” the timing, dosage, and clinical indication of GC therapy are the key points to better definitions in terms of treatment schedules according to different clinical settings in the future.

## The Timing of GC Therapy in Liver Failure

In the Asia-Pacific region, the most common type of liver failure is HBV-ACLF. The clinical stage of HBV-ACLF can be divided into four stages: early stage of ascending period, late stage of ascending period, platform period, and recovery period. Immune injury is the major event in the early stage of ascending period. The pathogenesis in the late stage of ascending period is involved in ischemia, immune injury, and hypoxia injury (25). During the platform period, body conditions achieve an immunosuppression state.

Endotoxemia is an important factor during the initiation of liver injury. Recent studies have shown that there was an inflammatory cascade in the early period of HBV-ACLF (26, 27). The sooner systemic inflammatory response syndromes (SIRS) occurred, the higher the mortality rate would be. GCs can inhibit inflammation, stabilize the liver cell membrane, and prevent further necrosis of liver cells (28). Therefore, early application of GC therapy can inhibit immune responses. The inhibition of systemic inflammation delays rapid progression and improves the survival rate of patients with ACLF.

Zhao et al. (11) found that patients responding best to GCs were those with less severe liver failure and a higher risk of rapid disease progression, with lower HE grades and MELD scores but extremely high ALT levels. The optimal time of intervention with GCs was within 14 days of the onset of symptoms.

We consider that the efficacy of GC treatment is primarily associated with the timing of GC administration. Meanwhile, the first-time physician, age, basic condition, and complications should also be considered for GC administration. Patients with some specified indicators can benefit more from GC therapy; these could be indicators such as ALT>1,000 U/L, TBIL in the 10~20 × ULN, PTA≥30%, MELD score <28, no obvious signs of infection, hepatic encephalopathy Stage < II, no liver and kidney syndrome trends, as well as overactive immunological responses. However, until now, there has been a lack of accurate quantitative indicators for GC therapy. Therefore, it is particularly important for doctors to accumulate more and more clinical experience.

## The Dose Selection for GC Therapy in Liver Failure

Today, the ideal choices regarding GC type and does remains inconclusive. Based on current clinical reports, GC dose is generally controlled in 1~2 g/kg/d (methylprednisolone). Kotoh et al. (23) explored the feasibility of large doses of GC treatment for the treatment of liver failure. They divided 34 patients with ALF into two groups; 17 patients were given methylprednisolone 1,000 mg/d via hepatic artery continuously for 3 days. As a result, 13 patients were cured, 2 patients died, and 2 patients underwent LT without serious complications. Fujiwara et al. (29) discussed the value of high-dose GCs in the treatment of HBV-related liver failure. It was found that the survival rate and liver regeneration in the GC-treated group showed a slim advantage, but there was no statistical difference, while patients with HBV infection and a poor basic condition had an unfavorable prognosis.

When the efficacy of GCs therapy cannot be determined in clinic, it is required that possible side effects of GCs are kept within a controllable range based on the principle of safety. GCs can significantly inhibit the presence of phagocytic cells to the antigen, promote the destruction and disintegration of lymphocytes, and develop the removal of lymphocytes from blood vessels so as to reduce the number of lymphocytes in circulation (30). Small doses of GCs mainly inhibit cellular immunity, while high doses of GCs can suppress humoral immune function by inhibiting B cells and antibody production (31).

The number of liver surface glucocorticoid receptors (GRs) may be reduced in liver failure (27). If greater doses of GCs are given, the GCs cannot play a role during the presence of receptor saturation, but may increase the incidence of side effects of GCs. Therefore, patients with liver failure, especially those with cirrhosis, are not recommended to use high-dose GCs. Although GCs can increase the incidence of infection and upper gastrointestinal bleeding, as well as other complications, the side effects of GCs are controllable. Therefore, it is essential to screen and monitor the side effects during GCs therapy in patients with liver failure.

## The Mechanism of the Potential Benefit of GC Therapy in Liver Failure

### The Core Pathogenesis of Liver Failure

Currently, it is widely accepted that “endotoxin-macrophage-cytokine storm” is the core pathogenesis of liver failure, combined with the immune injury as the initial factor in the development of liver failure, especially in the early stage of liver failure (27, 32).

The chemical essence of endotoxin is lipopolysaccharide [LPS, recognized by the pattern-recognition receptor toll-like receptor 4 (TLR4)] (33). With the interaction of LPS-binding proteins, it binds to a variety of cell membranes with receptor CD14, transmitting signals from the outside of the cell to nucleus and stimulating the synthesis and release of cytokines, which involves tumor necrosis factor-α (TNF-α), interferon-α (INF-α), IL-1, and IL-6 and simultaneously induces macrophages to secrete nitric oxide and large amounts of oxygen-free radicals (34–37). The liver cells are injured by delayed type hypersensitivity, oxidative stress, and apoptosis. If the immune response cannot be suspended in time, it would lead to a vicious cycle, resulting in significant liver cell necrosis, apoptosis, and liver failure (38–40). Peripheral blood mononuclear cells (PBMCs) and monocytes from patients with cirrhosis respond stronger to LPS stimulation (41). Heat shock proteins (HSPs) are well-known as protective proteins that make cells resistant to stress-induced cell damage. However, simultaneous activation of TLR4 by HSPs causes enhanced tissue injury (42).

Immune injury is considered as the first blow in the “triple hit theory” of liver failure, and timely suspension of its excessive immune response may reduce or even reverse its condition (43, 44). As the most commonly used anti-inflammatory and immunosuppressive agents, GCs can inhibit macrophage phagocytosis and antigen treatment and suppress the production of inflammatory cytokines. Therefore, GCs have the theoretical basis for the treatment of liver failure.

### The Anti-inflammatory Mechanisms of GCs

Aside from rapid non-genomic effects, GCs exert genomic effects by binding to the glucocorticoid receptor (GR), a member of the nuclear receptor family of transcription factors (45). Upon ligand binding, the GR translocates to the nucleus, where it acts either as a monomeric protein that affects transcription with other transcription factors or as a homodimeric transcription factor, which binds glucocorticoid response elements (GREs) in promoter regions of GC-inducible genes (46). Some reports have clearly showed that GR dimer-dependent transactivation is essential in the anti-inflammatory activities of GR (47–49). GR^dim/dim^ mutant mice were used to show reduced GR dimerization, and hence GC cannot control inflammation (50, 51).

## The Potential Mechanism for Controversial Actions of GC Therapy in Liver Failure

### The Pro-inflammatory Mechanisms of GC

Emerging studies have shown that GCs have a two-way regulation for inflammatory and immune responses (52). The basal state of the immune system and the type of exposure to GCs are significant factors influencing the effects of GCs (53). For instance, while chronic exposure to GCs seems to be immunosuppressive, acute exposure increases the peripheral immune response (54).

It was found that GCs can induce the expression of several innate immune-related genes, including several members of the Toll-like receptor (TLR) family, such as TLR2 and TLR4 (55–57). The activation of TLRs via the repression of NF-κB and AP-1 or via the induction of GC-induced leucine zipper (GILZ) or MKP-1 is a hallmark feature of inflammation (55).

The GR signaling interplays with the TLRs signaling pathway via several mechanisms (58). Hermoso et al. (59) found that dexamethasone increased TNF-α induction of TLR2 through the activation of GR, supporting the existence of positive feedback between the activation of the TLR signaling pathway and GC secretion. Meanwhile, GCs may exert pro-inflammatory actions through interactions with inflammatory cytokines such as TNF-α and acute phase protein serpinA3 (60). Besides, some studies indicated that GCs can work synergistically with pro-inflammatory mediators to enhance the defense mechanisms to ensure removal and clearance of pathogens in the hepatic acute-phase response (58, 61). GC-mediated activation of NLRP3, TLR2, and P2Y2R and the potentiation of LIF and TNF-α regulated pro-inflammatory genes (58, 62). All these results provide a potential explanation for the controversial actions of GC therapy in liver failure. More studies are required to characterize the liver-specific effects of the anti- and pro- inflammatory roles of GR signaling.

### GC Resistance (GCR)

There are two types of GCR, inherited or familial GCR and acquired GCR (63). It is accepted that a pro-inflammatory environment can negatively affect GR sensitivity (64, 65). The mechanisms contributing to reduced GC responsiveness are heterogeneous as they involve various cytokines and cell types. The mechanisms of GCR are still unclear. As GCR occurs in many inflammatory diseases, it is widely considered that GCR is a heterogeneous phenomenon with multiple underlying mechanisms (66). Some of these involve problems with the GR protein itself, but many others are independent from GR and involved in mutations in GR-induced genes and problems with chaperones or cofactors (63).

Meanwhile, the down-regulation of the GR protein is associated with GRC, involving many different mechanisms such as reduced transcription and homologous down-regulation (67), GR protein degradation (68), and decreased stability of GR mRNA (the involvement of AUUUA motifs in the 3′UTR of GR mRNA) (69). Moreover, post-translational modifications of GR also contribute to a reduced GC response, such as ubiquitination of the GR (Lys-426 within a PEST element) and phosphorylation of the GR (68, 70). Besides, some research also showed miRNAs have a prominent role in the regulation of GR mRNA turnover and the occurrence of GCR (71, 72).

In addition to the non-genomic and genomic actions of GCs, GR signaling also relies on the existence of post-translational modifications (PTMs) and multiple receptor isoforms. GR is transcribed from a single gene, NR3C1; however, alternative splicing of this gene generates GRα and GRβ isoforms (73). The GRβ isoform also participates in the GCR. It was found that up-regulation of the dominant negative GRβ isoform was correlated with GR insensitivity via inhibiting GR-induced transactivation and GR nuclear translocation (74).

### The Possible Factors of GC Refractoriness in Liver Failure: Sepsis

Sepsis is a common complication of ACLF, which is an acute systemic inflammatory disease (75). However, GCs are hardly useful in sepsis (63). Thus, sepsis is considered a GCR disease. GCR is an essential problem in sepsis and leads to: lack of transport and removal of bile acids in the liver, resulting in cholestasis; increased production and reduced removal of L-lactate, resulting in lactic acidosis; GCs having no anti-inflammatory effects.

Our previous study proposed that the diagnostic criteria of sepsis are not suitable for patients in HBV-ACLF with sepsis, because patients with underlying chronic liver disease and cirrhosis may have deranged clinical parameters (76). Therefore, it is essential to establish compatible diagnostic criteria for sepsis in patients with ACLF. When sepsis occurred, the serum TBiL level and WBC count elevated significantly while PLT count decreased significantly. We argue that when sepsis occurs during the process of liver failure GCs are not recommended for patients.

## Conclusions and Future Perspectives

The idea of using GCs during acute liver failure has circulated for so many years, but, so far, no meaningful work has provided conclusive evidence of its therapeutic efficacy, except in the field of autoimmune etiology. Beyond the east/west demarcation, current data availing GC's use in liver failure revealed benefits that appeared marginal and were no longer present upon adjustment (10), came from evidence recorded in non-randomized studies (22), or were other ones carried out in small groups of patients (23, 24). More results should be confirmed using a larger randomized clinical trial to in order to arrive at better definitions in terms of treatment schedules according to different clinical settings.

Meanwhile, due to the complicated pathophysiology of liver failure, the exploration of immunological manifestations with different etiology and different clinical staging of patients with liver failure is needed urgently. This is a prerequisite for the feasibility and safety of GC applications. With an in-depth study, we can find the accurate timing, dosage, and clinical indicators of GC therapy for the clinical management of liver failure, so that clinicians can make timely treatment options so as to obtain the greatest benefits for patients.

## Author Contributions

RX wrote this manuscript. QM designed this manuscript.

### Conflict of Interest

The authors declare that the research was conducted in the absence of any commercial or financial relationships that could be construed as a potential conflict of interest.

## Abbreviations

ALF: acute liver failure
GCs: glucocorticoids
HBV: hepatitis B virus
HBV-ACLF: hepatitis B virus-related acute-on-chronic liver failure
HE: hepatic encephalopathy
INF-α: interferon-α
LPS: lipopolysaccharide
SALF: subacute liver failure
SIRS: systemic inflammatory response syndromes
TNF-α: tumor necrosis factor-α.
